# Circulating biomarkers in the diagnosis and prognosis of immune checkpoint inhibitor-related myocarditis: time for a risk-based approach

**DOI:** 10.3389/fcvm.2024.1350585

**Published:** 2024-02-12

**Authors:** Gillian Murtagh, Christopher deFilippi, Qiong Zhao, Ana Barac

**Affiliations:** ^1^ Core Diagnostics, Abbott Laboratories, Abbott Park, IL, United States; ^2^ Inova Schar Heart and Vascular, Falls Church, VA, United States

**Keywords:** cardiotoxicity, biomarkers, myocarditis, immune checkpoint inhibitors, immuno-oncology

## Abstract

Immune checkpoint inhibitors (ICIs) are monoclonal antibodies that block immune checkpoints and therefore activate immune cells, allowing them to recognize and attack cancer cells. ICIs have revolutionized oncology practice, but their use has been complicated by immune-related adverse events (irAEs). Of cardiovascular (CV) irAEs, ICI-related myocarditis has received significant attention due to high mortality rates, ranging from 25% to 50%, despite its overall low incidence. Establishing the early diagnosis of ICI-myocarditis is important for early initiation of steroids and consideration of hospitalization in patients who are at risk for hemodynamic compromise and need high acuity care in a tertiary setting. In this review, we summarize the diagnostic and prognostic tools for ICI-myocarditis, including electrocardiography, echocardiography, cardiac magnetic resonance imaging, with emphasis on circulating biomarkers. Cardiac troponins (cTns) are an essential component of the diagnosis of ICI-myocarditis, and we provide a summary of the recent studies that utilized different assays (cTnI vs. cTnT) and outcomes (diagnosis vs. prognosis including major adverse cardiac outcomes). With the exponential increase in ICI use across different oncology indications, there is a major need to include biomarkers in risk stratification to guide diagnosis and treatment. Our review proposes a framework for future multisite registries, including cTn evaluation at baseline and at the time of irAE suspicion, with development of central biobanking to allow head-to-head evaluation and clinical validation of different biomarker assays in ICI-myocarditis. This approach, with the inclusion of CV biomarkers into clinical and pragmatic oncology trials, holds promise to improve the early recognition and management of ICI-myocarditis and CV irAEs, thus leading to better outcomes.

## 1 Immune checkpoint inhibitors in oncology and immune related adverse events

Immuno-oncology (IO) is a form of cancer treatment that utilizes the body's own immune system to recognize and target cancer cells. One of the key IO approaches involves the use of immune checkpoint inhibitors (ICIs). Immune checkpoints (ICs) are molecules present on the immune cells that regulate responses to antigens and in physiologic situations prevent immune system overactivation. Many cancers have the ability of binding to ICs to decrease the immune response and evade immune surveillance. ICIs are monoclonal antibodies that block the ICs and therefore activate immune cells, allowing them to recognize and attack cancer cells. Currently approved ICIs target two prominent IC pathways: (1) Programmed Cell Death Protein 1 (PD-1) signaling by binding and blocking PD-1 receptors (e.g., nivolumab, pembrolizumab, and cemiplimab) or PD-ligand 1 [(PD-L1), e.g., atezolizumab, avelumab, and durvalumab] and (2) Cytotoxic T-Lymphocyte-Associated Protein 4 (CTLA-4) pathways by binding and blocking CTLA-4 (e.g., ipilimumab).

ICIs have revolutionized oncology practice as multiple agents have been approved in treatment of different cancers in early, advanced, and metastatic settings (1). In 2022 there were more than 85 oncology indications for the 7 Food and Drug Administration (FDA)-approved antibodies targeting PD-1/PD-L11 pathways (2) and an analysis in 2019 indicated that more than a third of all patients with invasive cancer diagnoses in the US would be eligible to receive an ICI (3). The use of ICIs has been complicated by immune-related adverse events (irAEs) which result from overactivation of the immune system and may affect any organ and/or system. While irAEs differ widely in their clinical presentations, rapidly evolving and severe symptoms have been reported requiring prompt recognition and urgent treatment most often with steroids (4). Of cardiovascular (CV) irAEs, myocarditis has received the most attention due to its very high morbidity and reported mortality of 25%–50% in clinically symptomatic patients (5). The incidence of ICI-(related) myocarditis is low, ranging from 0.6% to 2.1% depending on the immunotherapy combination used, cancer type, and study design (5). The underlying mechanisms remain to be fully elucidated, but lymphocytic infiltrates in the myocardium point to T-cell mediated processes (6). Other CV events reported in ICI clinical trials have included pericardial disease, acute coronary syndrome (ACS), arrhythmias, and non-myocarditis related cardiac dysfunction (7) suggesting that different mechanisms may be underlying these clinical presentations. In this review we focus on ICI-myocarditis but emphasize the importance of the differential diagnoses and recognition of all irAEs.

## 2 Clinical presentation and diagnosis of ICI-myocarditis

Patients may present with a variety of symptoms including chest pain, dyspnea, fatigue, and/or palpitations, often mimicking ACS and/or heart failure (HF). Clinical features favoring ICI-myocarditis include recent initiation of ICI, most often within 30–60 days prior, and presence of other irAEs, (e.g., myositis, myasthenia gravis, pneumonitis, and/or hepatitis). The co-existence of severe myocarditis with myositis and/or myasthenia gravis has been reported (8, 9) and is recognized as clustered toxicity with recommendations for comprehensive evaluation when any one of the three conditions is found (10).

Currently recommended initial tests in patients with suspected ICI-myocarditis include electrocardiography (ECG), cardiac troponins (cTns), natriuretic peptides (NPs), and echocardiography (11). While there is general agreement about inclusion of these key clinical measures for the diagnosis of ICI-myocarditis (Table 1), there are significant variations in the definitions, reflecting the lack of high-quality data as well as the rapidly evolving field (10). Key characteristics of these diagnostic tests are summarized below followed by detailed discussion of circulating biomarkers.

**Table 1:** Examples of statements and society guideline criteria for diagnosis of myocarditis.

	ESC position statement on myocarditis (12)	ESC Cardio-Oncology guidelines (11) and IC-OS statement (13)	Proposed definitions for myocarditis in the setting of cancer therapeutics (14)
Clinical and diagnostic testing criteria	Clinical criteria • ACS-like • New or worsening HF • Chronic HF • Cardiogenic shock/Ventricular arrhythmia Diagnostic testing • ECG/Holter/stress test with new abnormalities • New LV or RV structural or functional abnormality (echo/angiography/CMR) • Myocardiocytolysis markers (elevated cTnI or cTnT) • Diagnostic CMR (edema and injury meeting Lake Louise criteria (15)	Pathohistological criteria • Multifocal inflammatory cell infiltrates with cardiomyocyte loss on biopsy/autopsy Clinical criteria Major criterion • Diagnostic CMR (meeting modified Lake Louise criteria (15) Minor criteria • Clinical syndrome^a^ • Ventricular arrhythmia and/or new conduction system disease • Decline in cardiac function, with or without RWMA • Other irAEs (particularly myositis, myopathy, myasthenia gravis) • Suggestive CMR (meeting some but not all of the modified Lake Louise criteria (15)	• Clinical syndrome of myocarditis Diagnostic testing Clinical criteria • ECG (evidence of myo-pericarditis) • Elevated biomarker of cardiac myonecrosis (cTn) • Echocardiogram (new RWMA) • CMR (meeting both (diagnostic) or some (suggestive) modified Lake Louise criteria (15) • Tissue pathology confirming myocarditis
To establish a diagnosis	Clinically suspected myocarditis: ≥1 clinical presentation + ≥1 diagnostic criteria from different categories, in the absence of: (1) CAD; (2) pre-existing CVD or extracardiac causes that could explain the syndrome. If patient is asymptomatic ≥ 2 diagnostic criteria should be met.	Pathohistological diagnosis or Clinical diagnosis (any of the following): • cTn elevation with 1 major criterion • cTn elevation with 2 minor criteria after exclusion of ACS and other causes	Definitive myocarditis (any of the following) 1. Tissue pathology 2. Diagnostic CMR + syndrome + 1 (ECG or cTn) 3. Echo RWMA + All (syndrome, cTn, ECG, exclusion of other diagnoses) Probable myocarditis • Diagnostic CMR (no syndrome, ECG, biomarker) • Suggestive CMR + syndrome, ECG, or biomarker • Echo RWMA + syndrome + biomarker or ECG • Syndrome + PET scan evidence and no alternative diagnosis Possible myocarditis • Suggestive CMR with no syndrome, ECG or biomarker • Echo RWMA + syndrome or ECG only • Biomarker + syndrome or ECG + no alternative diagnosis
Modifiers		• Severity (severe and non-severe) • Smoldering (without clinical symptoms) • Steroid-refractory • Recovery (recovering and recovered)	

ESC, European Society of Cardiology; IC-OS, International Cardio-Oncology Society; ACS, acute coronary syndrome; HF, heart failure; acute CAD, coronary artery disease; CVD, cardiovascular disease; cTn, cardiac troponin; LV, left ventricle; RV, right ventricle; RWMA, regional wall motion abnormalities; irAEs, immune-related adverse events; ECG, electrocardiogram; CMR, cardiac magnetic resonance; PET, positron emission tomography.

^a^ Clinical syndrome includes any of the following: fatigue, muscle weakness, myalgias, chest pain, diplopia, ptosis, shortness of breath, orthopnea, lower extremity edema, palpitations, lightheadedness/dizziness, syncope, cardiogenic shock.

## 3 Electrocardiography

A variety of ECG findings have been reported in patients with ICI-myocarditis, varying from life-threatening heart block, ventricular and atrial arrythmias and ST-elevation, to nonspecific ST-T wave abnormalities. ECG is usually the first test performed in a symptomatic patient and ECG abnormalities may overlap with those of ACS, requiring investigation of ischemia prior to being attributed to myocarditis. With regards to its prognostic value, retrospective analyses found associations between pathological Q-waves and mortality (16) and between QRS prolongation and major adverse CV events (MACE) (17) in patients with ICI-myocarditis.

## 4 Echocardiography

While reduced left ventricular systolic function and regional wall motion abnormalities (RWMA) on the echocardiogram can occur, a normal left ventricular ejection fraction (LVEF) has been demonstrated in more than 50% of patients with confirmed ICI-myocarditis, indicating that the presence of normal LVEF cannot exclude the diagnosis (18). In a retrospective analysis (19) including 140 patients with ICI-myocarditis, the presence of decreased global longitudinal strain (GLS) was a predictor of MACE regardless of LVEF (19); similar findings have been reported using global radial and circumferential strain (20)*.* Finally, in a surveillance study among 129 patients who received ICIs, a decline in GLS correlated with elevation in high sensitivity (hs) cTnI suggesting that GLS is associated with myocyte injury (21). Abnormal GLS is associated with multiple cardiac conditions (15) and echocardiography is not consistently performed in the baseline evaluation of patients receiving ICIs, thus assessing an interval decline in GLS may be challenging when toxicity is suspected. Therefore, further research into the role of GLS for risk stratification and diagnosis of ICI-myocarditis is needed.

## 5 Cardiac magnetic resonance (CMR) imaging

CMR is the gold standard imaging methodology for diagnosis of myocarditis, providing visualization of edema and inflammation. The modified Lake Louise criteria (22) require confirmation of an abnormality in T2-weighted images indicating myocardial edema (T2-based criterion), and T1-based criterion indicating myocardial injury (e.g., increased myocardial T1 map value, increased extracellular volume, or positive late gadolinium enhancement) to establish the CMR diagnosis of acute myocarditis. These cardiac imaging criteria have been incorporated into the International Cardio-Oncology Society (IC-OS) consensus statement on definitions of CV toxicities (13) and included in the European Society of Cardiology (ESC) Guidelines on cardio-oncology (11) as well as other documents (12, 14) (Table 1). In the ESC algorithm, diagnostic CMR constitutes a major clinical criterion and its presence in addition to elevation of cTn with an appropriate clinical scenario is diagnostic of ICI-myocarditis (11). However, the sensitivity of CMR criteria has been questioned in a study demonstrating that less than 30% of patients with confirmed ICI-myocarditis met Lake Louise criteria (23) leading to a recommendation that endomyocardial biopsy should be pursued in patients with negative CMR and clinical suspicion for ICI-myocarditis (10, 11). Among patients diagnosed with ICI-myocarditis, abnormal T1-values, quantitated by T1-mapping, were predictive of subsequent MACE, pointing to its potential role in risk stratification of these patients (24).

## 6 Circulating biomarkers

### 6.1 Cardiac troponins (cTn)

Though elevated cTn levels are considered necessary for the diagnosis of myocarditis, other etiologies that require immediate investigation must also be considered. The degree of elevation and presence or absence of a rising/falling pattern of cTnI and T provide important insights, as persistently elevated cTn is typically seen in ICI-related myocarditis, but rapid rising may be related to an ACS (25). Furthermore, cTnI and T levels are often many folds higher than the upper reference limit [(URL), typically defined by the manufacturer as the 99th percentile of a healthy general population]. Substantial variability has been noted based on the specific cTn assay and timing of sample procurement (26), however these issues have been difficult to reconcile given the low frequency myocarditis and heterogeneity of assays used in practice. Unique cut-offs still need to be validated to optimize negative predictive value (NPV) and higher thresholds that optimize positive predictive value (PPV) both at baseline and with serial assessments. Table 2 summarizes the literature by different clinical scenarios highlighted below.

**Table 2:** Summary: troponins for surveillance, diagnosis, and prognostication in ICI-myocarditis.

First author year	Study design	Surveillance (S)/ Diagnosis (D)/ Prognosis (P)	Total *n*	Endpoint(s) and definition of endpoint(s)	Myocarditis *n*^a^	MACE *n*	Tn assay	cTn threshold(s) and performance	Other notable biomarker findings
Coustal et al. (27)	Retrospective cross-sectional study	D	29	Myocarditis (13, 14)	29	157 deaths from myocarditis, 8 from cancer	NR	Site-specific ULNs Tn elevations 42-fold local ULN reported in the most severe cases, versus 3.6-fold in less severe (*p* = 0.001). Most severe (*n* = 11) vs. less severe (*n* = 18): 4.9-fold local ULN: sensitivity = 90.9%, specificity = 66.7%.	
Lehmann et al. (25)	Prospective cohort study	SD P	147: 60 cases,87 cases from registry	Myocarditis^a^ MACE: Sudden cardiac death, HF, ventricular arrhythmia, pacemaker implantation, respiratory failure	147	24	hs-cTnT (Roche) hs-cTnI (Siemens) hs-cTnI (Abbott) cTnI (Roche) cTnI (Siemens)	hs-cTnT was >URL in 23/23 patients, cTnI>URL in 17/19 and CK>URL in 16/22 within 72 h of first MACE Peak cTnT:URL day 1–3: AUC = 0.84 for MACE. cTnT:URL ≥ 32× was reported as most predictive of MACE (11.1 [95% CI, 3.2–38.0].	AUC = 0.70 for CK for MACE No assay/threshold details available for validation cohort.
Furukawa et al. (28)	Prospective observational study	S	126	Myocarditis (12)^b^	13, 4 with moderate-severe features.	NR	Hs-cTnI (Abbott)	hs-cTnI ≥ 26.2 ng/L and more than double the baseline. hs-cTnI > 26.2 ng/L in 18 patients of whom 13 had myocarditis as defined. No other thresholds reported.	All 4 patients with moderate to severe myocarditis had elevated CK preceding cTnI ≥ 26.2 ng/L.
Tamura et al. (21)	Retrospective cohort study	D	129	Myocarditis (12)^c^	6	1 death 1 cardiogenic shock	hs-cTnI (Abbott)	hs-cTnI ≥ 26.8 ng/L If the baseline hs-cTnI was >URL, twice the baseline level. 5/6 with myocarditis had elevated hs-cTnI at diagnosis. No other thresholds reported.	
Vasbinder et al. (29)	Observational cohort study	D	2,636	Myocarditis (14)	57: 27 from study population, 30 from an independent myocarditis cohort	1,212 deaths	hs-cTnT (Roche)	hs-cTnT ≥ 19 ng/L. 100% of myocarditis patients had hs-cTnT elevation at diagnosis. Median hs-cTnT level at the time of diagnosis was 393 ng/L (IQR: 110–1,323)	Each doubling in CK from baseline increased risk in incident myocarditis with HR: 1.83: 95% CI: 1.59–2.10; *P* = 0.007
Waliany et al. (30)	Prospective cohort study	S	214	Myocarditis (14)	3	1 death and 1 ischemic CVA	hs-cTnI (Siemens)	55 ng/L PPV 12.5% 1,000 ng/L PPV 75% 2,000ng/L PPV 100% (For myocarditis)	
Awadalla et al. (19)	Retrospective case control study (international registry)	D	193	Myocarditis (12, 18) MACE = CV death, cardiac arrest, cardiogenic shock, hemodynamically significant complete heart block.	101	51	NR	Site-specific ULNs^d^ Assay type/thresholds NR cTn levels in myocarditis: elevated in 98/101 Median value of 0.85 ng/dl (=8.5 ng/L) [IQR 0.17, 2.3] cTn in 59 controls <0.01 ng/dl (*p* < 0.001) (= 0.1 ng/l)	NT-proBNP elevated in 88% of cases, median 589 [IQR 208, 2,413] pg/ml. vs. 560 [IQR 243, 2,093] pg/ml in controls (*p* = 0.07)
Petricciuolo et al. (31)	Prospective observational study	NA	30	CV death/stroke/TIA/PE New-onset HF	NR	7 (2 CV deaths, 2 CVA/TIA, 3 HF)	hs-cTnT (Roche)	Baseline cTnT ≥ 14 ng/l AUC = 0.91 for the primary endpoint AUC = 0.8 for CV death 100% sensitivity, 73% specificity for primary endpoint 100% sensitivity for CV death, 59% specificity.	
Escudier et al. (32)	Retrospective cohort study	NA	30	Arrhythmia/conduction disorder, sudden cardiac death, PE, HF.	NR	30 Cardiotoxicity as defined 8 CV deaths	NR	Site-specific ULNs Assay type/thresholds NR cTn measured in 26/30 patients (87%); elevation reported in 46%	BNP elevated in 14/14 patients in whom data was available.

S, surveillance; D, diagnosis; P, prognosis; NR, not reported; MACE, major adverse cardiac events; Tn, troponin; cTn, cardiac troponin; hs-cTnT and hs-cTnI, high sensitivity cardiac troponin T and I; HF, heart failure; BNP, B-type natriuretic peptide; NTproBNP, N-terminal B-type natriuretic peptide; ULN, upper limit of normal; URL, upper reference limit; PPV, positive predictive value; IQR, interquartile ratio; HR, hazard ratio; CI, confidence intervals; CV, cardiovascular: CK, creatine kinase; CVA, cerebrovascular accident; TIA, transient ischemic attack; PE, pulmonary embolism; AUC, area under the curve.

^a^ Having at least a histological examination of cardiac biopsy specimens or cMRI consistent with myocarditis and presentation not explained by other conditions.

^b^ Elevated hs-cTnI +(1) ≥1 clinical presentation; (2) if asymptomatic, but ≥1 diagnostic criteria, including ECG/Holter/stress test features, functional and structural abnormalities on cardiac imaging/CMR.

^c^ Diagnosed as a pathological finding based on lymphocytic infiltration in the myocardium with myocyte loss.

^d^ For descriptive purposes, ULN and URL are used interchangeably but may differ per site-specific practice.

#### 6.1.1 cTn surveillance in asymptomatic patients

Screening for cardiac injury and assessing risk for subsequent symptomatic ICI-myocarditis could be feasible, but there are unique issues to this patient population. For example, a single institution study of prospective surveillance in 214 patients demonstrated the need to test 72 patients receiving ICI therapies to detect 1 case of myocarditis based on the hs-cTnI URL (30). The PPV at the URL (55 ng/L) was only 12.5% and a PPV of 75% required hs-cTnI threshold value of 1000 ng/L.

#### 6.1.2 cTn for diagnosis in symptomatic patients

cTn elevations of any extent have been reported in over 94% of patients with ICI-myocarditis (18, 21). A case series of 29 patients with ICI myocarditis reported elevations 42-fold the URL in severe cases, vs. 3.6-fold in less severe (27). In another study, cTn values correlated with myocardial histopathology and hs-cTnT values exceeding 300 ng/L (URL 19 ng/L) were found more frequently among patients with higher degrees of T-cell infiltration (33).

#### 6.1.3 cTn for prognosis of MACE in patients with ICI-myocarditis

In a multicenter study that investigated 35 patients with ICI-myocarditis, higher cTnT was associated with MACE, and 10-fold higher median cTnT values were reported in patients with MACE compared to patients without (1,450 vs. 140 ng/L, respectively) (18). In another study of patients with ICI-myocarditis, presence of elevated hs-cTnT:URL ratio of >32 within 3 days of presentation, was associated with a hazard ratio of 11 (95% CI, 3–38) for MACE (25). In this investigation, MACE definition included all myotoxicity with respiratory failure reported in 50% of patients with MACE, raising a question whether the prognostic value of cTnT may reflect its sensitivity to detect myotoxicity in addition to cardiotoxicity (25, 34). Supporting this hypothesis, mRNA expression of cTnT, but not cTnI, was found in the skeletal muscle in patients with ICI myositis (25) indicating a need for further research of clinical significance of cTnT in detecting and monitoring systemic myotoxicity.

#### 6.1.4 Clinical caveats for applying cTns in surveillance, diagnosis and prognostication of ICI-myocarditis

In general population cohorts, differences in associations with outcomes have been reported for low-grade elevations of hs-cTns: cTnI was associated with myocardial infarction (MI) and elevated cTnT was more strongly associated with all-cause mortality and non-CV death (35). Increased cTnT has also been found in the presence of skeletal muscle damage (36–38), which is similar to the observations in cardio-oncology literature where elevations in cTnT correlated with concomitant ICI-related myositis and myocarditis (25).

In patients with ICI-myocarditis, hs-cTnI has been noted to rise and fall more rapidly than cTnT (39), leading to recommendations for its preferential use in the initial assessment and diagnosis of ICI-myocarditis (10, 11), with cTnT having additional prognostic and potentially diagnostic utility for skeletal muscle myotoxicity.

The phenomenon of macrotroponin (macroTn) indicating formation of immunoglobulin-troponin complexes, is also coming to attention in the ICI population, where immune activation may result in autoantibody binding to circulating cTn, forming a macrocomplex. Initially considered a spurious cTn result finding, macroTn has been reported with all cTn assays and has been associated with myocarditis and cardiomyopathy (40–42). While clinical implications of macroTn in ICI-myocarditis remain an area of active investigation, elevated cTn must always be interpreted in conjunction with clinical context. If inconsistent, the laboratory should be consulted, as further analytic methodologies can be applied to investigate for macrocomplexes, and verification sought with another cTn assay.

### 6.2 Natriuretic peptides

Concomitant elevation of natriuretic peptides (B-type natriuretic peptide [BNP] and amino terminal proBNP [NT-proBNP]) is common in ICI-myocarditis. Elevated NT-proBNP was present in 88% of 83 patients with ICI-related myocarditis in one study (19), however NT-proBNP values were not significantly different in patients with subsequent MACE compared to patients without MACE. In another small surveillance study of 126 patients receiving ICI, BNP was elevated in 11 patients, in some possibly reflecting presence of baseline cardiomyopathy (28).

### 6.3 Creatine kinase

Elevations in CK and CK-MB have been utilized for diagnosis (29) and surveillance of ICI-myocarditis (28). Rising CK levels that predate elevations in cTns in ICI-myocarditis have been noted (28, 29), although CK and CK-MB are generally less sensitive and specific for myocardial injury. When peak biomarker levels measured within 3 days of admission in 57 patients with ICI-myocarditis were compared, hs-cTnT:URL was found superior to CK:URL ratio in predicting MACE 24.

## 7 Future directions

**Figure 1:**
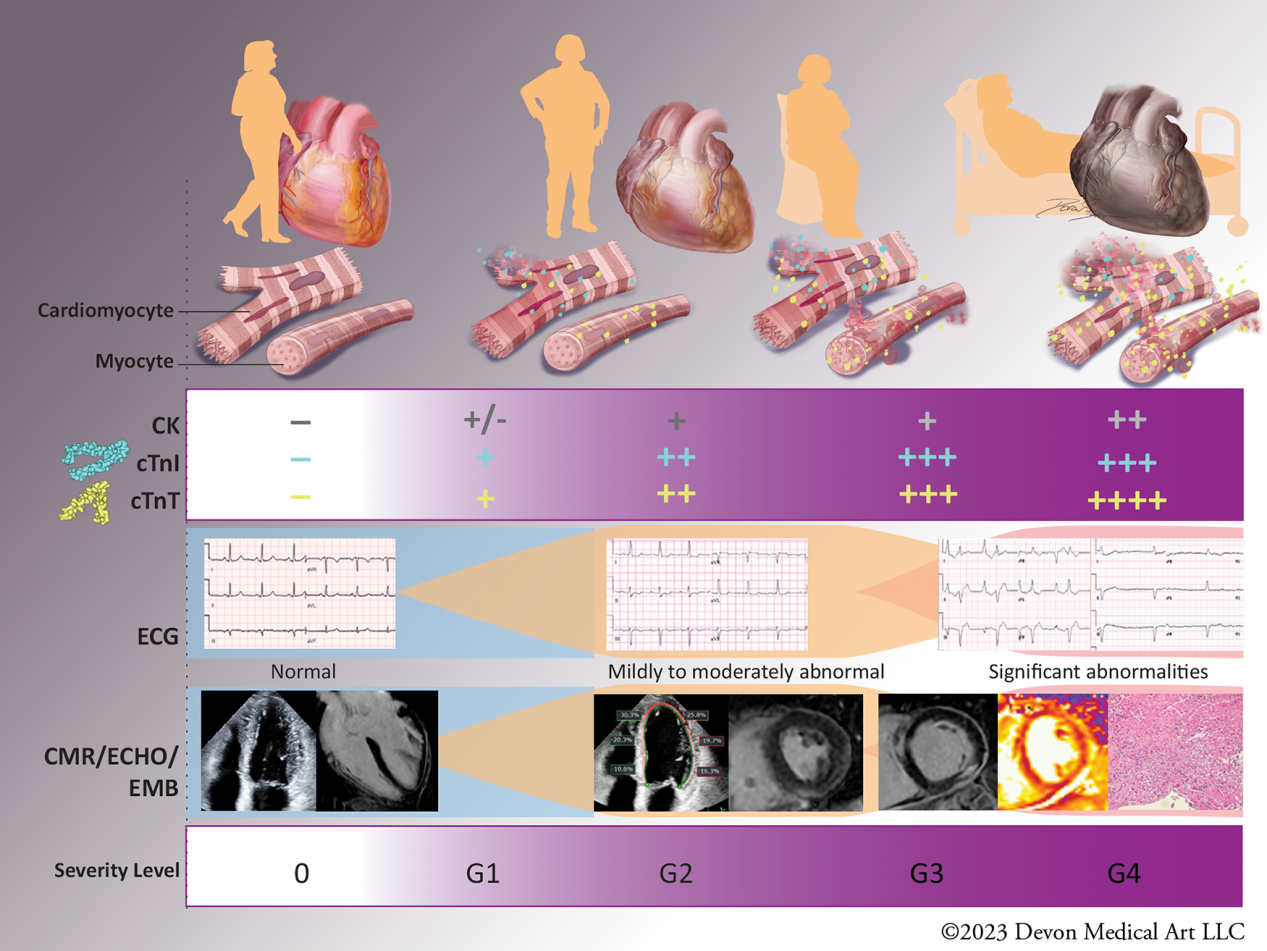
Biomarkers in diagnosis and severity of immune checkpoint inhibitor (ICI)-myocarditis. The diagnosis of ICI-myocarditis relies on clinical presentation, circulating biomarkers, cardiac imaging and endomyocardial biopsy in select cases. Mild elevations of cardiac troponins (cTns) have been described in asymptomatic patients without abnormalities in cardiac imaging (Grade 1, subclinical ICI-myocarditis), while patients with Grade 2 or mild ICI-myocarditis have abnormal cTns and some abnormalities on electrocardiogram (ECG), echocardiogram (ECHO), and/or cardiac magnetic resonance (CMR). Patients with moderate (Grade 3) or severe (Grade 4) ICI-myocarditis have clinical symptoms and often present with concomitant myositis reflected in increase in creatine kinase (CK) and cTnT. Severity of biomarker abnormalities has been shown to correlate with the adverse outcomes, however the exact cut-off values remain to be determined. “-”: values below the upper reference limit“; “+”: increments above the upper reference limit; CK, creatine kinase; hs-cTnI, high sensitivity cardiac troponin I; hs-cTnT, high sensitivity cardiac troponin T; ECG, electrocardiogram; ECHO, echocardiogram; CMR, cardiac magnetic resonance; EMB, endomyocardial biopsy; G1-4, Grade1-4.

With expanding indications for IO therapies, the need for accurate diagnosis of ICI-myocarditis and irAEs will continue to increase. Current diagnostic criteria rely on the detection of new diagnostic test abnormalities (e.g., new cTn increase, new RWMA, and/or new T1/T2 abnormality on CMR) which may be difficult to ascertain in absence of baseline values. The relevance of pre-treatment assessment is further emphasized in older individuals many of whom may have prior CV conditions, including MI or HF, and in whom differentiation of acute from chronic myocardial injury creates a particular challenge. At the present time baseline cardiac testing is not included in routine oncology practice (43) although it has been recommended in the ESC guidelines (11). Prospective studies evaluating circulating biomarkers at baseline (pre-treatment) and at the time of clinical suspicion are needed to further refine the diagnostic criteria and provide insight about the extent of myocardial injury in an individual patient (Figure 1). Adoption of different biomarker thresholds to identify risk for CV irAE may ultimately be needed for each assay, similar to requirements for the diagnosis of acute MI. This approach will also allow us to identify predictors of risk that should guide further diagnostic and treatment steps. In addition to cTns, CK and natriuretic peptides, novel biomarkers will be needed to elucidate the role of inflammation, metabolic and immune system derangement in pathogenesis of myocardial injury and other irAEs (44). Beyond ICI-myocarditis and irAEs, prospective investigations are needed to understand the association between ICI use and progression of atherosclerosis and plaque vulnerability which have been reported in the retrospective studies (45). Central biobanking of multisite registries incorporating baseline and serial sampling would allow more reliable, head-to-head evaluation of different cTn assays as well as investigation and clinical validation of novel biomarkers. Finally, inclusion of CV biomarker investigations into IO clinical and pragmatic trials holds promise to improve the early recognition and management of cardiotoxicity and lead to better outcomes.
